# Digital psychotherapeutic interventions to reduce dermatological symptom burden: Development and testing of MindMySkin

**DOI:** 10.1016/j.jdin.2025.09.017

**Published:** 2025-10-13

**Authors:** Jia Yi Chua, Valencia Long, Pei Ming Yeo, Jiayan Ding, Janet Dua, Nisha Suyien Chandran, Qasrina Lim, Hui Xin See Tow, Wilson Sim, Wan Ing Chong, Oliver Suendermann, Diego Pitta De Araujo, Jose Maria Valderas, Phillip Phan, Kean J. Hsu, Ellie Choi

**Affiliations:** a Department of Medicine, Yong Loo Lin School of Medicine, National University of Singapore, Singapore, Singapore; b Division of Dermatology, Department of Medicine, National University Hospital, Singapore, Singapore; c Sengkang General Hospital, Singapore, Singapore; d Department of Dermatology, Ng Teng Fong General Hospital, Singapore, Singapore; e Department of Internal Medicine, Singapore Health Services, Singapore, Singapore; f Department of Psychiatry, Ng Teng Fong General Hospital, Singapore, Singapore; g Intellect Pte Ltd, Singapore, Singapore; h Psychology, Faculty of Arts and Social Science, National University of Singapore, Singapore, Singapore; i Research Support Unit, National University Healthcare System, Singapore, Singapore; j Centre for Research in Health Systems Performance, Yong Loo Lin School of Medicine, National University of Singapore, Singapore, Singapore; k Department of Family Medicine, National University Health System, Singapore, Singapore; l Carey Business School, Johns Hopkins University, Baltimore, Maryland; m Department of Medicine, Johns Hopkins University, Baltimore, Maryland; n Mind Science Centre, Yong Loo Lin School of Medicine, National University of Singapore, Singapore, Singapore

**Keywords:** behavioral, chronic skin disease, complex interventions, dermatology, digital, eczema, feasibility study, health services research, implementation, mental health, mhealth, patient outcomes, patient-centered care, psoriasis, psychodermatology, psychological, psychotherapeutics, urticaria, user acceptability

## Abstract

**Background:**

Although cognitive and behavioral factors significantly impact dermatological outcomes, psychobehavioral interventions remain inaccessible and underused.

**Objective:**

To develop and test a psychobehavioral intervention (MindMySkin) delivered via a mobile application.

**Methods:**

Needs assessment, evidence-based module development, and formative user-centered evaluation were conducted in participants with eczema, psoriasis, or chronic urticaria. The developed prototype modules were further iteratively refined following additional qualitative and quantitative feedback.

**Results:**

27 participants contributed to 24 1:1 interviews and 231 responses between June 2023 and November 2024. Modules were highly rated (median score 4 or 5/5) for utility, interest, understandability, and recommendation for inclusion, underscoring strong acceptability. Participants valued the informative content and conversational tone but highlighted skepticism toward reflection exercises and oversimplification of patient difficulties. Design challenges include balancing time demanding but effective modules with shorter more appealing content. The final intervention comprised 74 modules addressing illness coherence, symptom management, functional impairment, emotional impairment, and the patient–physician relationship.

**Limitations:**

No participant reviewed the full program and modules were assigned, limiting insights on voluntary adherence.

**Conclusion:**

This study establishes the unmet need for psychotherapeutic support and demonstrates the acceptability, appropriateness, and usability of a self-administered psychobehavioral intervention in improving dermatological care.

**Capsule Summary**

• Psychological interventions are important yet underused in managing chronic inflammatory skin diseases.
• This study develops and demonstrates the feasibility of MindMySkin, a digital psychotherapeutic intervention suitable for use alongside clinical care.

## Introduction

Skin conditions are among the leading causes of disability globally,^1^, ^2^, ^3^ with quality of life (QoL) impairment expected to further increase.^4^^,^^5^ Unlike other chronic diseases, in many skin conditions, the correlation between QoL impairment and objective disease severity is weak.^6^^,^^7^ Instead, QoL impairment has a stronger correlation with psycho-behavioral factors such as anxiety, depression, resilience, coping, and body image.^7^, ^8^, ^9^

Studies have demonstrated the effectiveness of psychological interventions, including habit reversal training, relaxation techniques, and cognitive behavioral therapies, in decreasing the disease burden of inflammatory skin diseases.^10^, ^11^, ^12^, ^13^, ^14^, ^15^ Despite the evidence, such interventions are not routinely prescribed, partially because dermatologists lack the time and training to deliver them and instead prioritize biomedical therapeutics.^8^^,^^15^^,^^16^

Digital self-administered interventions, such as mental health applications, have emerged to meet this gap by offering a scalable solution that functions independently of active clinician intervention.^17^^,^^18^ When well constructed, these can extend the therapeutic reach of dermatologists, psychiatrists, and psychologists beyond traditional clinical settings, allowing specialists to focus on patients with more complex needs. However, as little as 3% to 4% of available applications are supported by scientific evidence or even affiliated with healthcare experts.^19^ This underscores a very clear need for the structured development and rigorous evaluation of such digital interventions.

**Fig 1:**
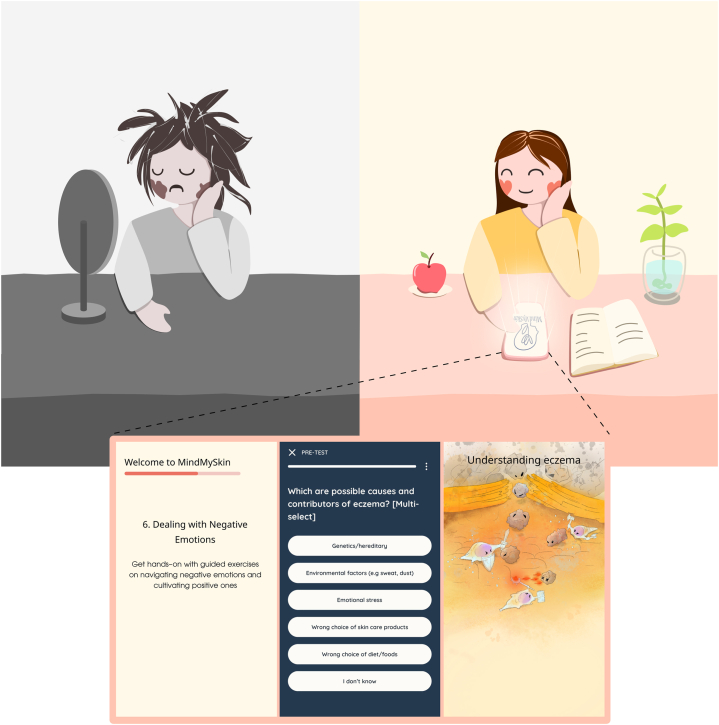
Graphical depiction of MindMySkin’s purpose.

In response, we developed MindMySkin, a digital self-administered psycho-behavioral intervention deployed via a mobile application. The purpose of this framework-guided intervention is to target unhealthy psychological, behavioral, cognitive, and social habits to improve self-efficacy, resilience, and coping skills, thereby reducing the symptom burden and impact of skin disease on QoL (Fig 1).

## Methods

We used the UK Medical Research Council Framework for Developing Complex Interventions^20^ to guide our development process and adapted it with specific modifications to accommodate nuances of digital intervention development. The multidisciplinary team consisted of dermatologists (V.L., N.C., and Y.P.M.), psychodermatologists (E.C. and J.D.), a clinical psychologist (K.H.), a cognitive behavioural therapy (CBT) trained counselor (C.W.I.), a psychiatrist (C.H.), behavioral economist (P.P.), industry partner (Intellect Inc), and patient representatives (J.C., A.C., and R.L.). Patient representatives were involved at the start of development and played a pivotal role in ensuring that the content aligned with patients’ interests and perspectives and providing comments about the study design and questionnaires. The National Healthcare Group Domain Specific Review Board approved this study (2022/00751).

The development of the intervention included the following stages.

### Needs assessment

Relevant patient factors were identified from our previous studies.^7^^,^^8^ They included realistic self-assessment of disease severity, knowledge about the disease, dealing with disease chronicity, tolerating distress, healthy disease representations, social comparisons,^21^, ^22^, ^23^ destructive self-talk, scratching, itch, self-control, self-efficacy, concerns about topical steroids and medication adherence,^24^, ^25^, ^26^ maintaining normal social life, and effective communication with the physician.^27^^,^^28^

In addition, we conducted semi-structured qualitative interviews with 24 participants (between June 2023 and January 2024) to gather feedback on their challenges, needs, and desired features of a mobile application. Inclusion criteria included individuals aged 16 years and older diagnosed with eczema, psoriasis, or urticaria of a minimum 3-month duration. Participants were recruited via convenience sampling from outpatient clinics or word of mouth. 1:1 interviews were conducted in person or over Zoom, and transcripts were analyzed using inductive thematic analysis, where codes and themes were allowed to develop with progressive and iterative analysis of data.

### Co-design and crafting of the content

We began the process with a literature review in December 2022 on existing psychotherapeutic interventions for dermatological disease, such as cognitive-behavioral therapy and habit reversal training, which have been shown to be effective in the dermatological population.^11^, ^12^, ^13^^,^^17^ Strategies addressing related conditions such as chronic pain, fatigue, and health anxiety were also reviewed.^29^, ^30^, ^31^, ^32^, ^33^

We then used the Theory of Change model^34^^,^^35^ as a guiding framework to systematically map the logical pathway linking patients’ needs, psychotherapeutic interventions, intermediate outcomes, and long-term outcomes. Existing psychotherapeutic strategies such as cognitive behavior therapy, habit reversal training, and guided imagery^14^^,^^36^^,^^37^ were adapted to address each identified need, such as itch, visible skin lesions, disease chronicity, and cyclicity.^38^ Where existing strategies were lacking, we developed new ones using the COM-B model and Behavioral Change Wheel^39^ such as distraction, social comparison and self-talk to manage needs such as low self-efficacy, emotional distress, and activity limitation.

Content was formatted into 4 major categories: (1) Learning paths for delivery of core concepts, (2) Guided journals for self-reflection and symptom tracking, (3) Rescue sessions for quick relief during physical or emotional distress, and (4) Therapeutic audio tracks for mindfulness, relaxation, and grounding. The intervention also included self-assessment tools such as quizzes and questionnaires to monitor disease severity, emotional well-being, and coping patterns.

This started in March 2023 and was followed by multiple rounds of iteration, testing, refinement (c.f. point 3), and was finalized in January 2025. The team leveraged its combined strengths and experience in developing educational content, designing behavioral health interventions, and promoting the adoption of digital health tools.^25^^,^^40^, ^41^, ^42^, ^43^, ^44^, ^45^, ^46^, ^47^ Simultaneously, our application development collaborator brought industry expertise in optimizing user engagement and mobile health platforms.^18^^,^^48^^,^^49^ These aimed to optimize the intervention for digital delivery to ensure accessibility, engagement, and ease of use while maintaining its core therapeutic principles.

### Formative user-centered evaluation

The developed content was tested with participants, with the inclusion criteria and recruitment strategy remaining identical to those outlined before, and allowing for the possibility of overlapping participants (modules could be tested as part of the qualitative interviews and each participant could test more than 1 module).

Participants were individually presented with Word or PDF copies of the content and asked to provide structured and open-ended feedback for each module. Structured quantitative feedback was collected using a 5-point Likert scale ranging from 1 (strongly disagree) to 5 (strongly agree) on constructs including the module’s level of interest, ease of understanding, usefulness, time required for completion, and recommendation for including that module in the application. Open-ended qualitative feedback was also gathered.

Each module was tested by at least 3 participants. Modules were often retested iteratively following edits prompted by negative feedback or significant suggestions from participants. Modules that consistently scored low (with an average rating of 3/5 or lower) were dropped from the intervention.

## Results/Findings

**Fig 2:**
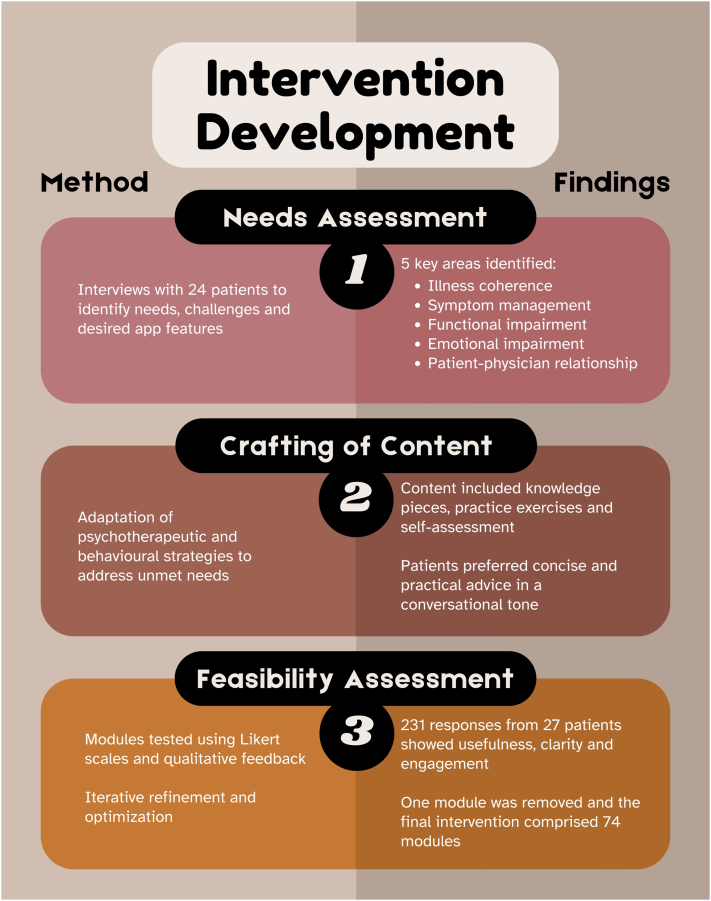
Key methods and results of the study’s 3 stages.

Fig 2 presents a summary of the 3 stages, which are further elaborated below.

### Identification of patients’ needs

Five key areas of need were identified from 24 qualitative interviews, namely, illness coherence, symptom management, functional impairment, emotional impairment, and the patient–healthcare provider interaction. Within these, subthemes include difficulty understanding and accepting disease chronicity and unexplainable flares, challenges managing itch and scratching, limitations with hobbies and daily activities, negative emotions of helplessness and self-pity, rushed consultation with physicians, and inability to promptly obtain medical advice during flares (Supplementary Table I, available via Mendeley at https://data.mendeley.com/datasets/b5vwjypnt2/1).

### Development of the intervention

#### Informational content

Table I presents a Theory of Change Model^35^ which details the interventional content, showing how the proposed solutions attempt to solve the identified needs and the intended outcomes and measures for evaluation. Naming and grouping of the behavior change techniques follows the Behavior Change Technique Taxonomy by Michie et al.^50^

**Table I:** Relationship between identified need, proposed solution, intended outcome, and outcome measure (Theory of Change Model)

Clinical need	Interventional content proposed	Mechanism of action∗	Proposed outcome measure
Illness coherence			
Information gaps leading to inability to understand and accept condition Lack of understanding of cause, chronicity, natural course of condition, and therapy	Learning paths Improving understanding and management of eczema, psoriasis, and chronic urticaria - Comprehensive educational resource covering disease pathogenesis, triggers, idea of chronicity, and misconception. A guide to topical steroids - Appropriate and safe usage of topical steroids and nonsteroidal alternatives.	Shaping knowledge	Questions assessing content knowledge (only in-application) IPQ-R Acceptance of chronic health conditions scale Adherence scale (validation in progress)
Inaccurate assessment of severity Perceiving higher severity than clinically determined.	Learning paths 1. Evaluating the severity of your skin condition - Teach disease rating for better self-assessment including estimating BSA affected - Illustrate disease severity through photographs	Exposure to entire spectrum of disease severity, improving nature of social comparison	Difference between patient and physicians’ rating of global disease severity. Confidence of self-assessment in disease severity (only in-application)
Symptom management			
Managing itch Inability to control scratching Distressing itch crisis Impaired sleep from itch	Learning paths 1. Itch neuroscience education 2. How to reduce itching and scratching Guided journal 1. Characterizing your scratching Rescue session 1. Overcoming intense itching attacks Mindfulness sessions - Guided audio including progressive muscle relaxation, hypnotic suggestions, and guided imagery	Shaping knowledge, goals and planning, feedback and monitoring, natural consequences repetition and substitution, and regulation antecedents	Itch, sleep, and pain NRS 5-D itch scale (only in-application) PROMIS itch (only in-application) DLQI (QoL impairment) WPAI Physician-assessed scratching scale
Challenges with a cyclical disease Suboptimal and reactive management	Guided journal 1. Written action plan 2. Weekly check in 3. Trigger journaling	Shaping knowledge, repetition, and substitution	IPQ-R Brief Resilience Scale DLQI
Functional impairment			
Activity limitations Difficulties with function and sleep	Learning paths 1. Values training and gratitude list 2. Tips for adapting to a life with skin disease 3. Time to work out our problems	Associations repetition and substitution Antecedents Identity	WPAI DLQI Adherence scale
Interpersonal and social interactions	Learning paths 1. Dealing with unwanted comments 2. Improving appearances	Valued self-identity	DLQI, PROMIS Self-Efficacy for Managing Emotions 4a, depression 8b
Emotional impairment			
Negative thoughts Sense of helplessness Negative cycle of rumination, self-pity	Learning paths 1. Creating space from negative thoughts - Cognitive defusion techniques 2. Trading negative thoughts for balanced insights - Cognitive restructuring	Shaping knowledge, comparison of behavior, associations, and regulation	IPQ-R BRS PROMIS Depression 8b and Anxiety 7a
Negative emotions Feeling ‘upset’ and ‘depressed’ Believing happiness depends on curing their skin condition	Rescue sessions 1. Tackling the worry 2. STOPping our negative emotions Guided journal 1. Emotion and gratitude journaling 2. Self-care and gratitude Mindfulness audios 1. Sitting with discomfort	Nonspecific incentive, framing/reframing, valued self-identity, and self-belief	IPQ-R WHO-5 PROMIS Self-Efficacy for Managing Emotions 4a, Depression 8b, and Anxiety 7a
Low self-esteem and body image Negative social comparisons to others	Learning paths 1. Befriending yourself 2. The link between skin conditions and body image 3. What (I think) people think about me 4. Dealing with unwanted comments 5. Improving appearances 6. Embracing body dissatisfaction & living well Mindfulness audios 1. Noting - Letting go of comparisons	Framing/reframing, valued self-identity, and self-belief	WBIS DLQI WHO-5 DSC
The patient–healthcare provider interaction			
Insufficient time and rushed consult	Learning paths 1. How to achieve the most from your consult	Shaping knowledge Improved communication	Partners in health (only in-application) DLQI
Assumptions† Patients will recognize the value of the content and engage with the application Users are able to comprehend the English language and can navigate a health application.			
External factors‡: Competition from other mobile applications and mental health tools, cyber-security threats or data breaches, possible vendor withdrawal			

IPQ-R encompasses domains including illness coherence, emotional representation, consequences, cyclicity, personal, and treatment control domain.

*5-D*, 5-dimensional; *BRS*, Brief Resilience Scale; *BSA*, body surface area; *DLQI*, Dermatology Quality Life Index; *DSC*, Dermatology Social Comparison; *IPQ-R*, Revised Illness Perception Questionnaire; *NRS*, Numerical Rating Scale; *PROMIS*, Patient-Reported Outcomes Measurement Information System; *QoL*, quality of life; *WBIS*, Weight Bias Internalization Scale; *WHO-5*, World Health Organization-Five Well-Being Index; *WPAI*, Work Productivity and Activity Impairment Questionnaire.

∗ Terminology follows the Behavior Change Technique Taxonomy by Michie et al (2013).

† Assumptions refer to underlying beliefs considered necessary for the intervention to be successful.

‡ External factors are factors beyond control that can facilitate or impair success.

The following approach was used to encompass an integrated and comprehensive management approach encompassing both biological and psychosocial dimensions.

1. Disseminating knowledge via text, illustrations, and audio recordings within structured modular learning paths.
2. Employing pictorial storylines to enhance relatability and peer learning.
3. Theoretical education strengthened with practical walk-through exercises on psychological tools like cognitive behavioral therapy and habit reversal training.
4. Using questionnaires with score interpretation and reference banding, encouraging awareness and self-insight.
5. Offering guided journaling to facilitate recollection, reflection, and monitoring.
6. Providing rescue sessions with distraction or mindfulness techniques to ameliorate distressing symptoms like itch.

#### Content delivery and user experience

As short, easily digestible and enjoyable content are important elements for today’s users of digital applications;^51^^,^^52^ the content strategy included breaking down information into brief concise pieces. Relatable quotes and the use of nonconfrontational language were incorporated to improve engagement. We avoided oversimplifying explanations with metaphors or lay terms, choosing to retain the scientific names of medications and treatments for reliability while maintaining a conversational and professional tone. This decision promotes transparency and empowers users by fostering a better understanding and management of their treatment plans.

While many applications focus on gamification for engagement, we wanted to avoid trivializing content or creating additional distraction and cognitive load.^53^^,^^54^ Instead, we focused on creating an interface that minimizes clicks and provides seamless navigation between modules to enhance engagement.

#### Platform and technical development

MindMySkin was hosted onto *Intellect*, an existing mobile application developed by Singapore-based Intellect Inc.^55^ The Health Insurance Portability and Accountability Act (HIPAA)-compliant application offers mental health strategies targeted at the general population with proven efficacy in prior randomized trials on stress reduction,^48^ worry,^18^ and body image.^49^ Existing modules of the application were excluded from present study; however, we leveraged Intellect’s expertise in content development and existing infrastructure for cost-effective and synergistic integration.

The application employs a robust technical stack, including Amazon Web Services for secure cloud computing, React Native for responsive cross-platform functionality, and MySQL for efficient data management. *Intellect* is ISO 27001 certified, compliant with Singapore's Personal Data Protection Act and employs Zero-Knowledge Encryption, encrypting data at the device level for user-only access.

### Formative user-centered evaluation

A total of 231 responses were collected from 27 participants between June 2023 and November 2024. Median feedback scores ranged from 4 to 5 on a Likert scale (5 being the highest), suggesting high ratings for the usefulness, clarity, and engagement of the modules (Table II). Time commitment was also deemed reasonable and there was strong support for including the modules in the intervention (median score of 5; interquartile range: 4-5).

**Table II:** Summary of structured feedback from formative evaluation (*n* = 231)

Domains	Median score (interquartile range)
Utility	4 (4-5)
Interest	4 (4-5)
Time requirement∗	5 (4-5)
Understandability	5 (4-5)
Coherence/sensibility	5 (4-5)
Insensitivity	Yes: 7.36%; No: 92.64%
Recommendation for inclusion	5 (4-5)

Responses were graded on a 5-point Likert scale ranging 1 (strongly disagree) to 5 (strongly agree).

∗ Two different statements were used for this domain: “The length of the meditation session was reasonable” for mindfulness sessions (median = 4 [4-5]), and “The time taken to complete the content/tool was reasonable” for all other modules (median = 5 [4-5]). Responses were combined.

Common themes from the qualitative feedback included appreciation of the ‘informative’ content that were ‘easily digestible’ and ‘short and sweet’ (Table III). Longer modules that took 10-15 mins were viewed by some as too lengthy. At the same time, we recognized the need to cultivate increased attention and for sufficient time commitment to facilitate meaningful learning. Thus, we retained these modules and exercises while reducing the quantity of text as far as possible without compromising learning.

**Table III:** Summary of open-ended qualitative feedback from formative evaluation

Theme	Exemplar quote
Likes	
Knowledge dissemination in understandable format	“Bite-sized nuggets of information… easy to digest.” “Would not have considered behavioral and psychological aspects to my condition if not for these questions. Good insight to how I feel.” “Adds to knowledge - learned something new, more than what doctors will tell patients.”
Practical and relevant solutions	“Gives practical solutions and alternative methods to reduce scratching.”
Enhancing self-awareness, resilience, and emotional well-being	“Helps a patient to reflect on our inner emotions and allows us to be sure of what we are feeling.” “Makes the user feel like there’s a friend to talk to and there’s some guidance in processing through the negative thought.” “Helps you to see how to coexist with your skin condition”.
Dislikes	
Too much reflection, too little actionable solutions	“What is the point of grading yourself on a scale if nothing comes out of it in the app? Too much self-reflection.” “Doesn't provide methods to break away from the thought patterns.”
Privacy and data security concerns	“Patients need to know that the photos submitted will be kept confidential…not sure who will be seeing it or what it could be used for.”
Lack of visual aids in patient education	“More visuals for understanding things related to your own body.” “The instructions could be better followed with a cartoon diagram. Or a gif of someone doing it.”
Lack of human interaction and limited responsiveness to user needs	“Those looking for help, there is no follow through. Eg who to talk to.” “A counseling hotline might be better than facing a screen… Won’t feel heard in an app.”
Areas of confusion or insensitivity	
Challenging to understand the mind-skin connection	“Hard to visualize how the improved mind actually helps with the skin.”
Excessive text and overuse of medical jargon	“A lot more illustrations/pictures should be included. Reading a wall of text is very boring.” “Some of the text seems very doctor-centric.”
Unrelatable, toxic positivity, and oversimplification of complex issues	“The comic strip was pretty insensitive…as if to illustrate that as long as they have body positive imagery, everything would be fine... Having such a simplified cartoon of 2 juxtapositions won't be relatable to anyone, because more often than not, eczema embodies both states at once.”
Disempowering advice, teaching tolerance over boundary setting	“The module teaches responding politely to unwanted comments...in a way that feels like patients owe kindness to people…make it so the content helps the patient to navigate uncomfortable situations rather than teaching them how to appear more palatable to the public.”
Other comments	
Time consuming	“Feels long, may not have patience to finish it.”
Impersonal advice	“I do not feel that it helps me because it asks me to focus on my own issues without asking for the help that I need.”
Desire for real-time human guidance and interaction	“I would suggest having someone to guide people through this app…without any human guidance, can be a bit confusing.” “Vocalizing my thoughts would flag to medical caregivers that I have an issue and also allow a better rationalization of them.”
Desire for peer support and community engagement	“[Meditation] has its limitations as there is no subsequent step with an active approach…. A community approach could be done…might benefit better from sharing and learning from other patients who walked the path, for emotions, ailments, and strategies.” “The negative emotions from patients should be channeled into something positive to help others, either to share their pain or to inspire them in the journey of healing.”
Desire for engaging user interface and more visual aids	“I think that the interface really matters a lot…having a simple and intuitive interface.” “Visuals could be added to keep the users attention.”

Some participants expressed preference for practical and actionable solutions over reflective exercises for insight building and wanted to see a clearer rationale for the psychologically focused modules. In response, we simultaneously reduced the volume of reflective questions asked as part of the intervention and provided data-backed explanations underscoring the relevance of building self-awareness and emotional coping strategies.

Areas highlighted as being potentially insensitive were carefully reviewed. One area related to the oversimplification of the challenges patients faced. For example, some participants felt that being asked to relax, reframe their thought, or focus on beneficial solutions were pressuring them into positivity and was not something they could control. Changes were made to emphasize patient difficulties and to acknowledge progress as a continuum rather than a dichotomy. For example, goals were framed as achieving balanced rather than purely positive thoughts, and mindfulness techniques were designed to foster acceptance without judgment rather than the pursuit of wellbeing per se.

A conversational tone such as “Hi, it’s me, Dr…” was well received for both text and audio files. Participants expressed acceptance and in some cases, a preference for recordings by local study team members over professional voice actors, even if the narration was less polished. Textual information was copyedited to balance engagement (eg, use of emojis, simple and catching language) with professionalism and scientific rigor.

In the process of testing, modules were extensively edited and reorganized. Various new modules were added while 1 module, a guided food journal, was removed as most participants found this overly tedious and of little value (Table IV). The final intervention comprised 74 modules, each taking an average of 7.2 (standard deviation: 4.0) minutes to complete. These were organized into the 5 key needs earlier identified: illness coherence, symptom management, functional impairment, emotional impairment, and the patient–physician relationship.

**Table IV:** Examples of changes made from formative evaluation

Area of improvement	Changes made
Poor engagement	Shortened and simplified text, minimized jargon, adopted a conversational tone, added emojis, and additional illustrations. Removed module on “guided food journal” and streamlined tracking of triggers which was deemed tedious.
Preference for more practical solutions and less reflection	Reduced reflective questions and added clear explanations on how psychological strategies benefit skin health. Interspersed greater practical tips and concrete recommendations among reflective exercises.
Insensitive advice (toxic positivity and disempowering)	Refined content to be more nuanced and reflective of patient-lived experiences, avoided simplistic advice, acknowledged individual differences in handling challenges, and offered options for solutions.
Customizable user experience	Modules made optional and accessible at any time.
Peer support and community	Added quotes on patient-lived experiences and links to local skin support groups.

## Discussion

Symptom burden comprises the interplay between objective disease and the cognitive and behavioral responses of the patient^56^, ^57^, ^58^ and both need to be addressed. The development of MindMySkin represents a theoretically grounded and evidence-informed attempt to rigorously develop and deliver structured psychodermatological care. Early involvement of stakeholders such as patients and commercial partners optimizes it for sustainable adoption while plans for subsequent testing in a randomized controlled trial^59^ will provide efficacy data.

Lessons that can be drawn for developers of patient education include the need to balance differing objectives of what the clinician wants to deliver, what the patient actually needs for improvement, and what the patient finds enjoyable and meaningful. For example, patients in our study tended to prefer actionable and tangible solutions for specific symptoms, while the clinical team wanted to also promote introspective elements with a focus on coping and self-management. Explaining the rationale of learning different skills (mindfulness and balanced thinking) was understood to be important but challenging to convey in written words.

Another challenge was optimizing and personalizing the content for each user. The same feature was simultaneously enjoyed by some and disliked by others. Some valued the focus on emotional well-being, while others considered those modules “too soft”. Repetition, which is crucial for building a skill, was perceived as some by redundant and excessive. To accommodate differing preferences, we provided module suggestions throughout the intervention to guide participants to suitable modules while keeping all modules optional and freely accessible. Future use of detailed algorithms and artificial intelligence could enable greater personalization.

Limitations include use of convenience sampling, which skewed the sample toward younger participants with higher literacy, although this arguably aligns with the intervention’s target population. Additionally, each participant appraised only selected modules over a relatively short period (module-level feasibility), which reduced participant burden but meant that no participant had reviewed the entire set of modules. Additionally, the long-term adherence and voluntary use of the application without pressure or reminders from the study team remain to be seen. Finally, the intervention currently has limited integration with clinical care pathways and electronic health records which could improve usability and utility.

## Conflicts of interest

Author E.C. has received grant funding from Pfizer for an investigator-initiated trial on atopic dermatitis, that is unrelated to present work. She is also associate/assistant editor for Journal of the American Acaedemy of Dermatology (JAAD), JAAD International, and International journal of Dermatology. Author P.P. holds editorial positions for the International Journal for Quality in Health Care, Medicine, and Journal of Technology Transfer. Author N.S.C. has received fees for participation in advisory boards from AbbVie, Johnson & Johnson, Sanofi, Pfizer, DKSH, L'Oreal and Novartis, investigator fees for clinical trials from AbbVie, Novartis, Amgen, Sanofi, and Boehringher Ingelheim and speaker honoraria from Galderma, Johnson & Johnson, LEO, Pfizer, Sanofi, and Lion Corporation. Author O.S. is an employee of Intellect Pte Ltd, a mental healthcare company based in Singapore, and hold equity in the company. He contributed valuable industry insights for the content. However, he had no involvement in the study design, collection, analysis of data, patient interactions, the determination of the final intervention content, or decision to publish. He participated in writing and reviewing of the manuscript. Intellect Pte Ltd does not hold any copyright or ownership rights to the MindMySkin application. All authors have played a role in the development of MindMySkin. There are no other conflicts of interest relevant to this study to declare.
